# The effects of chronic high-dose morphine on microgliosis and the microglial transcriptome in rat spinal cord

**DOI:** 10.1177/17448069231183902

**Published:** 2023-06-26

**Authors:** Fredrik HG Ahlström, Hanna Viisanen, Leena Karhinen, Kert Mätlik, Kim J Blomqvist, Tuomas O Lilius, Yulia A Sidorova, Vinko Palada, Pekka V Rauhala, Eija A Kalso

**Affiliations:** 1 Faculty of Medicine, Department of Pharmacology, 60655, University of Helsinki, Helsinki, Finland; 2 Faculty of Medicine, Individualized Drug Therapy Research Programme, 60655, University of Helsinki, Helsinki, Finland; 3 Department of Clinical Pharmacology, 3836, University of Helsinki and Helsinki University Hospital, Helsinki, Finland; 4 Department of Emergency Medicine and Services, 3836, University of Helsinki and HUS Helsinki University Hospital, Helsinki, Finland; 5 Laboratory of Molecular Neuroscience, Institute of Biotechnology, HiLIFE, 3835, University of Helsinki, Helsinki, Finland; 6 Faculty of Medicine, Department of Physiology, 3835, University of Helsinki, Helsinki, Finland; 7 Faculty of Medicine, SleepWell Research Programme, 60655, University of Helsinki, Helsinki, Finland; 8 Department of Anaesthesiology, Intensive Care Medicine and Pain Medicine, 3836, Helsinki University Hospital and University of Helsinki, Helsinki, Finland

**Keywords:** Opioids, microglia, transcriptomics, rat, neuroinflammation, apoptosis, circadian rhythm

## Abstract

**Background:** Opioids are efficacious and safe analgesic drugs in short-term use for acute pain but chronic use can lead to tolerance and dependence. Opioid-induced microglial activation may contribute to the development of tolerance and this process may differ between males and females. A link is suggested between this microglial activation and inflammation, disturbances of circadian rhythms, and neurotoxic effects. We set out to further delineate the effects of chronic morphine on pain behaviour, microglial and neuronal staining, and the transcriptome of spinal microglia, to better understand the role of microglia in the consequences of long-term high-dose opioid administration. **Experimental Approach:** In two experiments, we administered increasing subcutaneous doses of morphine hydrochloride or saline to male and female rats. Thermal nociception was assessed with the tail flick and hot plate tests. In Experiment I, spinal cord (SC) samples were prepared for immunohistochemical staining for microglial and neuronal markers. In Experiment II, the transcriptome of microglia from the lumbar SC was analysed. **Key Results:** Female and male rats had similar antinociceptive responses to morphine and developed similar antinociceptive tolerance to thermal stimuli following chronic increasing high doses of s.c. morphine. The area of microglial IBA1-staining in SC decreased after 2 weeks of morphine administration in both sexes. Following morphine treatment, the differentially expressed genes identified in the microglial transcriptome included ones related to the circadian rhythm*,* apoptosis, and immune system processes. **Conclusions:** Female and male rats showed similar pain behaviour following chronic high doses of morphine. This was associated with decreased staining of spinal microglia, suggesting either decreased activation or apoptosis. High-dose morphine administration also associated with several changes in gene expression in SC microglia, e.g., those related to the circadian rhythm (*Per2, Per3, Dbp*). These changes should be considered in the clinical consequences of long-term high-dose administration of opioids.

## Introduction

Opioids are among the most efficacious drugs for the treatment of severe nociceptive pain. They exert their effect primarily through μ-opioid receptors (MOR) in the dorsal horn of the SC, the periaqueductal grey (PAG), and the rostroventral medulla (RVM).^1^ They are, however, less effective in treating neuropathic pain (NP)^2^ and have several use-limiting adverse effects. Tolerance, opioid-induced hyperalgesia (OIH), and dependence are potential problems when opioids are used for long periods.^3^ Tolerance and OIH are mediated through several mechanisms, including sequestration of β-arrestin-2^4,5^ and microglial activation.^6–8^

Microglia are the principal immune cells of the central nervous system (CNS),^9^ and they react to several insults, including spinal cord trauma and ischaemic stroke.^10,11^ Also, their role in neurodegenerative diseases such as Parkinson’s and Alzheimer’s disease has been extensively studied.^12^ Their role in the development and maintenance of neuropathic pain has received increasing attention.^13^ In essence, together with other glial cells, they maintain the homeostasis of the CNS. Our understanding of the function of these cells has changed from that of passive surveyors to active modulators of the CNS.

As such, it is not surprising, that an increasing body of evidence suggests other consequences of opioid therapy, including disturbances in circadian rhythms and neuroinflammation due to microglial activation.^6,14–19^ These phenomena may in turn be linked, as microglial cells display circadian rhythmicity in inflammatory responses^20–23^ and microglial ablation shifts the circadian rhythms of rats towards the inactive phase.^24^

High-dose opioid administration may also cause apoptosis through neurotoxic effects in patients.^25–28^ High-dose chronic oxycodone has been shown to activate pro-apoptotic pathways and neuronal axonal degeneration in rats^29^, possibly through MORs, which microglia may express.^8,18^ NMDAR-dependent activation of caspases by morphine has also been described to lead to apoptosis.^30^ In vitro, morphine has been shown to cause apoptosis in human microglia and neurons.^31^ However, the significance of opioid-related neuronal or glial apoptosis is not established.

Adding to the complexity of the role of microglia in opioid therapy and pain, previous data point to significant differences between the sexes in pain behaviour and mechanisms.^32^ A recent study showed that while the knockout of microglial MORs delayed the development of tolerance in both male and female mice, OIH was abolished in males only.^8^ Also, in NP, the roles of microglia and other components of the immune system seem to differ between the two sexes.^33–35^

A better understanding of the long-term effects of opioids is crucial for several patient populations, including opioid abusers and cancer patients using high daily doses of opioids for pain management. As microglial cells may be implicated in these processes, and an increasing amount of evidence points to differing roles for these cells depending on the sex, we set out to study the transcriptomes of isolated SC microglia in both male and female rats following a high-dose morphine administration regimen. We also characterised changes in pain behaviour and conducted immunohistochemical analyses of the expression of microglial IBA1 and neuronal NeuN markers in the lumbar region of the SC.

## Materials and methods

### Study protocol

**Figure 1.**
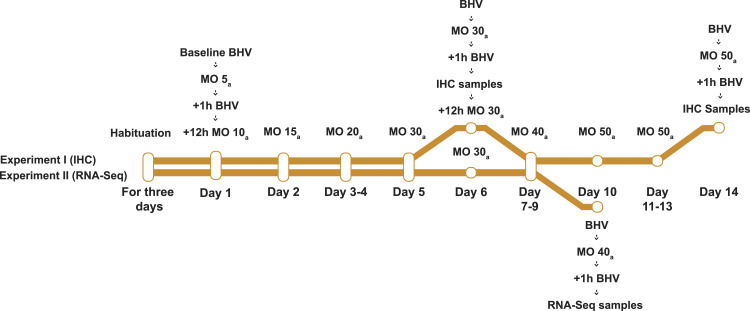
Study protocol and morphine treatment schedule. In both experiments, baseline behaviour was measured after habituation for 3 days. Then, animals received either morphine hydrochloride, with dose given in mg/kg (MO) or saline s.c, every 12 h indicated by subscript _a_. One hour after the injection, pain behaviour (BHV) was registered with hot-plate and tail-flick tests. After this, injections continued twice daily for 6 or 14 days in Experiment I and for 10 days in Experiment II. Pain behaviour was again measured after 6 and 14 days in Experiment I and after 10 days in Experiment II, before and one hour after injections. Spinal cord samples were collected for immunohistochemistry (IHC) on Days 6 and 14 in Experiment I, and on Day 10 for RNA-Seq in Experiment II.

Our study comprises two individual experiments (Experiment I and II), where rats were chronically administered morphine hydrochloride and their pain behaviour was tested (Figure 1). In Experiment I, upon completion of behavioural tests, we collected paraformaldehyde-perfused (PFA) SC samples for immunohistochemistry (IHC). In Experiment II, microglia were extracted from the SC for RNA sequencing at the end of the experiment. Experiment I continued for up to 14 days, but some animals were killed for sample collection for IHC already on day 6. Experiment II lasted for 10 days.

### Animals

Experiments I and II made use of 48 and 32 Sprague-Dawley rats, half male and female, respectively. The average weights of the male and female animals at baseline were 206 and 185 g, respectively. Animals were housed in individually ventilated cages, with two of the same sex in each cage, at the Laboratory Animal Centre of Biomedicum, University of Helsinki. Standard food and water were freely available; the light and dark periods were 12 h each. All treatment groups were habituated in a similar fashion for 3 days prior to the experiment. Stress to the animals was minimized. The ARRIVE guidelines were followed during the study and the 3R principle was applied to minimize harm to the animals. The Finnish Act on the Protection of Animals Used for Scientific or Educational Purposes (497/2013) and the EU Directive 2010/63/EU on the protection of animals used for scientific purposes were followed. The Regional State Administrative Agency for Southern Finland approved all animal experiments conducted (ESAVI-9697/04.10.07-2017).

### Morphine treatment and behavioural testing

Animals were randomized into equal-sized morphine and saline groups; the researchers giving injections and performing behavioural tests were blinded as to treatment groups.

Morphine (administered as the hydrochloride, mass calculated as free morphine) or saline solution was injected twice daily subcutaneously (s.c), with increasing doses in the morphine group for the duration of both experiments. Doses were increased slowly due to the respiratory depression caused by opioids. Morphine was diluted in saline, with a consistent 1 mL/kg injection volume. In both experiments, morphine doses increased from an initial 5 mg/kg, as shown in Figure 1. The final doses were 30 mg/kg on Day 6, 50 mg/kg on Day 14 (Experiment I), and 40 mg/kg on Day 10 (Experiment II). Protocols using similar maximum doses have previously been used.^36^ One hour after the last injections, the final behavioural tests were conducted, and samples were extracted.

To assess changes in pain-related behaviour following acute and chronic morphine administration, we used tail-flick and hot-plate tests to measure thermal nociception.^37^ These tests were conducted at baseline (before commencing injections), after 6 and 14 days of chronic administration in Experiment I, and after 10 days of chronic administration in Experiment II. Testing was repeated on the aforementioned days 1 hour after morphine or saline injection to determine the acute changes in behaviour due to morphine treatment.

#### Tail flick test

After 10 minutes of habituation in plexiglass tubes, animals underwent three cycles of testing, from which the mean time to a flick was calculated for each. The time required from heat stimulus to flick was recorded. Testing was conducted with the Tail Flick 37360 apparatus (Ugo Basile, Italy). A cutoff time of 10 s was used to avoid tissue damage, should no response be elicited.

#### Hot plate test

After the tailflick test, the hot plate test was conducted with the rat placed in a transparent circular tube on a Hot Cold Plate 35100 apparatus (Ugo Basile, Italy). The temperature was set to 52°C. A shaking or licking of the hind paw, or jumping, was counted as a positive response, after which the animal was removed, and the time to response was recorded. A 60 s cutoff time was imposed in case the animal did not exhibit a response.

### Sample collection

In Experiment I, lumbar spinal cord (L4) samples were collected after 6 and 14 days upon completing the behavioural tests. On these days, PFA-perfused samples of SCs were collected. All animals were first transcardially perfused with PBS for 3 minutes under isoflurane anaesthesia,^38^ after which perfusion with 4% PFA was carried out for 15 min. After excision, PFA-perfused tissues were submerged in 4% PFA solution, which was changed after one and 3 days. After this, samples were embedded in paraffin blocks.

In Experiment II, lumbar microglial RNA samples were extracted, as previously described.^39^ L4-L5 regions of SCs were carefully collected and placed in Hank’s Balanced Salt Solution (HBSS) containing no Ca^2+^ or Mg^2+^ ions (#14170112, ThermoFisher, United States). The tissue was dissociated using the Neural Tissue Dissociation Kit (P) (#130-092-628, Miltenyi Biotec, Germany) and gentleMACS™ Dissociator (Miltenyi Biotec, Germany), according to the manufacturer’s instructions. The cell suspension was filtered through 70 μm cell strainer and pelleted by centrifugation (300 × g; 10 min). For myelin removal, the cell pellet was resuspended in 400 μL of 0.5% BSA (#A3294, Sigma, United States) in phosphate-buffered saline (PBS/BSA), mixed with 10 mL of 30% isotonic Percoll solution (#GE17-0891-01, Sigma, United States) and centrifuged for 10 min at 680 × g, decelerating without braking. The pellet was resuspended in HBSS (#H8264, Sigma, United States), pelleted at 300 × g for 10 min and resuspended in 80 μL PBS/BSA, supplemented with 2 mm EDTA. After the addition of 12 μL of anti-CD11b-coupled magnetic microbeads (#130-093-634, Miltenyi Biotec, Germany), the cell suspension was incubated at +4°C for 15 min, washed with 2 mL of PBS/BSA and cells pelleted at 300 × g for 10 min. The pellet was resuspended in 0.5 mL of PBS/BSA and the cell suspension applied to an LS column (#130-042-401, Miltenyi Biotec, Germany) placed on a QuadroMACS Separator. After washing with 9 mL of PBS/BSA, the column was removed from the magnet and magnetically labelled cells washed out with 5 mL of PBS/BSA. The cells were collected by centrifugation and used for RNA isolation.

### Immunohistochemistry with antibodies against IBA1

Lumbar segments of SCs were postfixed in 4% PFA in PBS for at least 24 h and embedded in paraffin blocks. Sections of 10 μm thickness were prepared from the samples.

SC sections were probed with antibodies for IBA1 (1:1000, Cat# 019-19741, FUJIFILM Wako Pure Chemical Corporation, United States). Bound antibodies were visualized using anti-rabbit biotinylated secondary antibodies and the VECTASTAIN ABC HRP Kit (Cat PK-6101, PK-4002 Vector Laboratories, United States) with 3,3′-diaminobenzidine (DAB) as a chromogene (according to manufacturer’s instructions). Labelled slides were dehydrated and mounted, using Coverquick 2000 (Q PATH, Cat#05547530). After drying, the slides were imaged using the 3DHISTECH Scanner (3DHISTECH Ltd, Hungary) at Biocenter Helsinki (http://www.biocenter.helsinki.fi/bi/histoscanner/index.html).

The number of IBA1-positive cells and the area covered by immunopositive staining were determined as the total number of pixels with intensity above the selected threshold. These were normalized to the area of analysed tissue in pixels in the left and right dorsal and ventral horns and quantified using scripts developed in-house with manually set size and intensity thresholds, as described previously.^39^ For each animal and region studied, two non-consecutive sections were quantified at first. If the results for these two sections differed by more than 50%, two to four additional sections were analysed. For statistical analysis, the data for each animal were averaged.

### Immunofluorescence with antibodies against NeuN

Lumbar segments were prepared as with the samples stained for IBA1. The SC samples were stained with antibodies against NeuN (1:170, 14H6L24, Invitrogen, United States). Secondary Alexa 488 fluorescent-labelled antibodies were used for visualization with immunofluorescence (IF) microscopy. Labelled slides were dehydrated and mounted using Coverquick 2000 (Q PATH, Cat#05547530). The slides were imaged using the Zeiss Axio Imager 2 with Apotome, at Biomedicum Imaging Unit (Biomedicum Helsinki) (https://www2.helsinki.fi/en/infrastructures/bioimaging/biu/instruments/widefield-microscopes#section-39215). We imaged sections of the white matter of the SC of identical sizes and quantified the percentage of area stained, using an automatically determined image-specific threshold in Fiji software.^40^ For each animal, two sections were quantified, and the results for each animal were averaged.

### RNA sequencing

RNA from CD11b^+^ cells was isolated with the TRIzol^TM^ reagent (#15596026, ThermoFisher, United Arares). SMARTer Stranded Total RNA-Seq Kit v2- Pico Input Mammalian (Takara, Japan) kit was used to generate cDNA libraries for RNA sequencing (according to manufacturer’s instructions). Total RNA (10 ng) was fragmented and converted to cDNA. Library quality was assessed by Bioanalyzer (Agilent DNA High Sensitivity chip) and library quantity by Qubit (Invitrogen). Paired-end sequencing was performed with the NextSeq High Output 1 × 75 bp flow cell Illumina NextSeq 500 instrument. The Functional Genomics Unit at Biomedicum Helsinki carried out the differential expression analysis. Sample quality was assessed with the FastQC package.^41^ Trimmomatic software was used for light quality trimming.^42^ Subsequently, reads were aligned to the Ensembl.org Rnor_6.0 genome^43^ with STAR.^44^ FeatureCounts was used to make count tables.^45^ Finally, differential expression was calculated using the DESeq2 package,^46^ with significant differential expression defined as false discovery rate (FDR) value <0.05.

### Statistical analysis

Statistical analyses of the behavioural data and IHC/IF data were carried out using Graphpad Prism 8 (GraphPad Software, United States). Comparisons were made using multiple t-tests or two-way ANOVA analyses, with corrections for multiple comparisons, where applicable. Pathway analyses were conducted with iPathwayGuide v1906^47^ (AdvaitaBio, United States)

As detailed previously,^48^ clustering of the genes with significant up- and downregulated expressions was performed using Gene Cluster 3.0 software with heatmap visualizations were produced using Java TreeView 1.16r4 software.

## Results

### Behavioural tests

**Figure 2.**
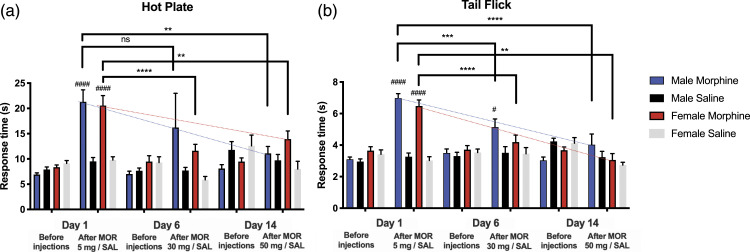
Responses to thermal nociceptive stimulation in female and male rats following chronic, twice daily with increasing doses, and acute administration of s.c. morphine in Experiment I. The first dose on Day 1 was 5 mg/kg, on Day 6 (before testing the effects of morphine) 30 mg/kg, and on Day 14, 50 mg/kg. Results from the hot plate test and the tail-flick test are shown in ((a) and (b)), respectively. Mean response times and SEM are reported; 8-12 rats per group. Two-way ANOVA with Holm-Sidak correction: **p < 0.01, ***p < 0.001, ****p < 0.0001, as shown over the square brackets. Dashed lines illustrate the trend in response times in the morphine groups; hashes indicate same-day comparisons, before and after morphine: #*p* < 0.05, ####*p* < 0.0001.

In Experiment I, in the hot plate test on Day 1, response times increased significantly in both sexes 1 hour after morphine injections (5 mg/kg s.c.), compared with saline (*p* < 0.0001) (Figure 2(a)). On Day 6, (30 mg/kg s.c.), and on Day 14 (50 mg/kg s.c.), morphine did not increase response times in males or females. On Days 6 and 14, the response times 1 hour after morphine (30 mg/kg s.c. and 50 mg/kg s.c.) administration had decreased compared with Day 1 1 hour after morphine (5 mg/kg) in females (Day 6 *p* < 0.0001, Day 14 *p* = 0.0040), while in males, this decrease was seen only on Day 14 (*p* = 0.0042), but not on Day 6.

In Experiment I, on Day 1, tail flick response times at 1 hour after morphine administration (5 mg/kg s.c.), increased significantly in both sexes compared with saline (*p* < 0.0001) (Figure 2(b)). On Day 6, tail flick response times 1 hour after morphine (30 mg/kg s.c.) had decreased significantly compared with Day 1 in females, with no difference between the morphine and saline groups on Day 6. In males, on Day 6, response times in the morphine group were still significantly longer than in the saline group (*p* = 0.0159), but shorter than on Day 1, one hour after morphine (*p* = 0.0003). On Day 14, morphine (50 mg/kg s.c.) did not increase the response times 1 hour after the injection, in either sex, suggesting antinociceptive tolerance.

**Figure 3.**
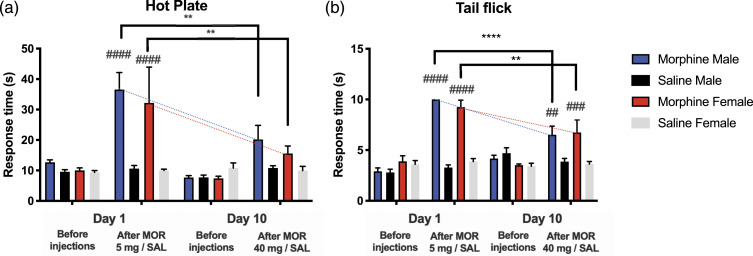
Responses to thermal nociception in female and male rats following chronic, twice daily administered increasing doses, and acute morphine administration in Experiment II. The first dose of morphine on day 1 was 5 mg/kg and the dose on day 10 was 40 mg/kg. All injections were subcutaneous. Results from the hot plate test and the tail flick test in ((a) and (b)), respectively. Mean response times and SEM are reported; eight rats per group. Two-way ANOVA with Holm-Sidak correction: **p < 0.01, ****p < 0.0001, as shown over the square brackets. Dashed lines illustrate the trend in response times in the morphine groups; hashes indicate the same-day comparisons, before and after morphine: ##*p* < 0.01, ###*p* < 0.001 ####*p* < 0.0001.

In Experiment II, in the hot plate test on Day 1, in both sexes, response times increased significantly 1 hour after the morphine injections (5 mg/kg s.c.), compared with saline (*p* < 0.0001) (Figure 3(a)). On Day 10 (40 mg/kg s.c.), both female and male response times had decreased significantly 1 hour after morphine, compared with Day 1 after morphine (male *p* = 0.0055, Female *p* = 0.0048). There was no difference on Day 10 after morphine and saline in either sex.

In Experiment II, both sexes showed increased response times in the tail flick test 1 hour after the first injection of morphine (5 mg/kg s.c.) (*p* < 0.0001) (Figure 3(b)). On Day 10, compared with Day 1, tail flick response times 1 hour after morphine injection (40 mg/kg s.c.) had decreased significantly in both sexes (Male *p* < 0.0001, Female *p* = 0.0043); however, a difference between the morphine and saline groups were still observed in both sexes on Day 10 (Male *p* = 0.0024, Female *p* = 0.0002).

Chronic morphine administration did not affect response times measured before the injections, in either sex, in either tail flick or hot plate test, on any day in Experiment I or II (Figures 2 and 3).

At no time point was there a significant difference between males and females in either the hot-plate or tail-flick response times in either Experiment I or II (Figures 2 and 3).

Saline did not change the hot plate or tail flick response times in any comparison in either sex group in tests prior to or after saline administration in Experiment I or II. (Figures 2 and 3).

### Immunohistochemistry and immunofluorescence

**Figure 4.**
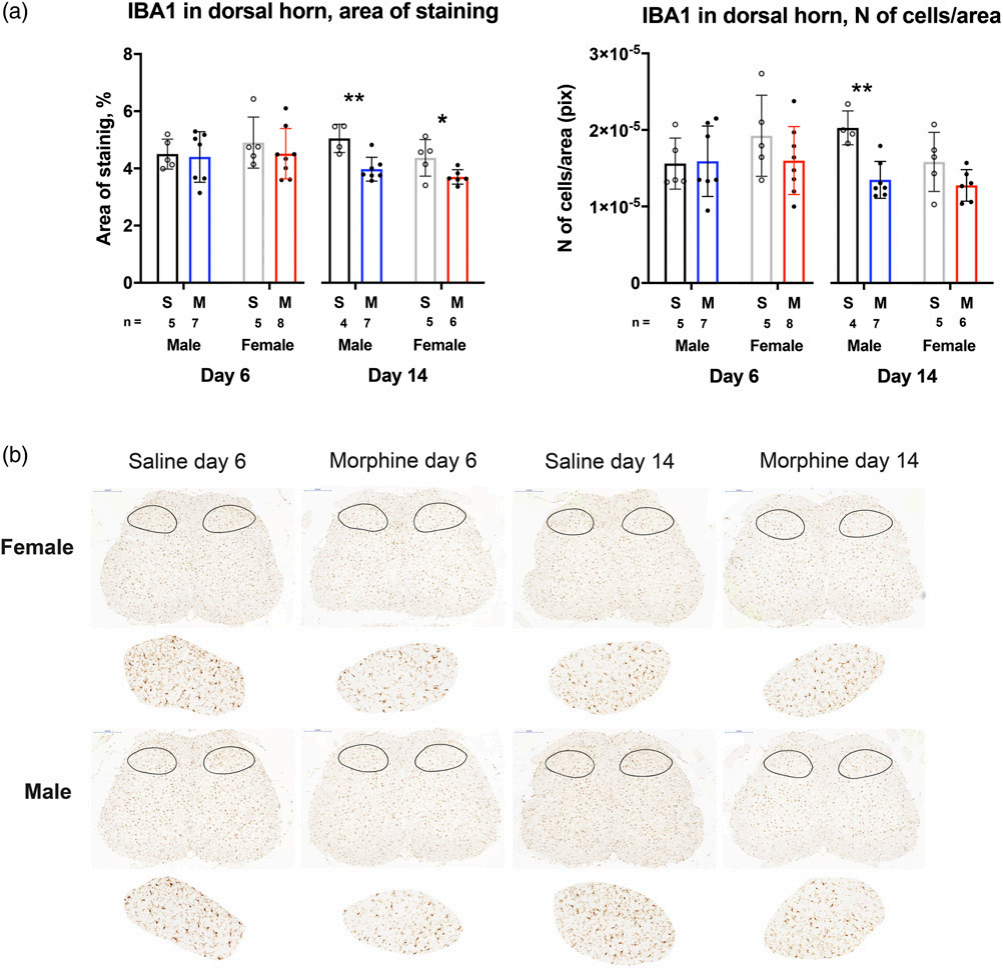
Analysis of IBA1-positive staining cells in the dorsal horn of the L4 spinal cord (SC) of male and female rats following chronic morphine administration on Days 6 (up to 30 mg/kg s.c.) and 14 (up to 50 mg/kg s.c.). Area of staining and number of stained cells per area were calculated, and mean and SEM are reported in (a). Representative images of the whole lumbar spinal cord and with dorsal horn indicated by black outline are shown in (b), with representative analyzed dorsal horn sections displayed below (approx. ×2.5 magnification compared with the whole spinal cord images). *p < 0.05, **p < 0.01, same-day morphine (M) and saline (S) groups of the same sex were compared. Student's*t* test with Holm-Sidak; Male saline day 6 *n* = 5, Male morphine day 6 *n* = 7, Female saline day 6 *n* = 5, Female morphine day 6 *n* = 8, Male saline day 14 *n* = 4, Male morphine day 14 *n* = 7, Female saline day 14 *n* = 5, Female morphine day 14 *n* = 6; N of cells/area = number of cells/area.

Samples of lumbar SCs stained for number of IBA1-positive cells and area of staining of IBA1 in the dorsal horn demonstrated no change in either sex on Day 6, when the morphine group was compared with the saline group (*p* > 0.05) (Figure 4). On Day 14, however, the area of staining had decreased in both male and female morphine groups, compared with the saline group (M: *p* < 0.01; F: *p* < 0.05); and the number of cells per area had decreased in males, but not in females, (M: *p* < 0.01; F: *p* > 0.05). Representative images of the stained areas are found in Figure 4.

**Figure 5.**
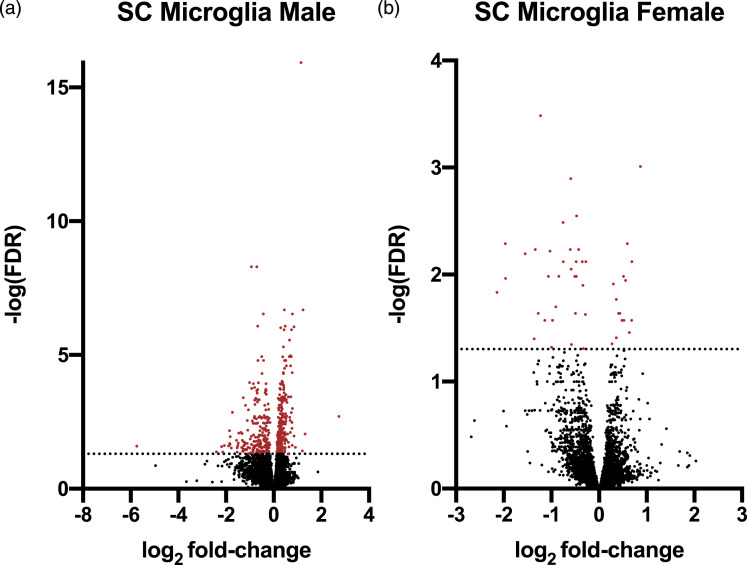
Volcano plots of transcriptome in rat spinal cord (SC) microglia following chronic high-dose morphine administration. Volcano plots, for males and females in ((a) and (b)) respectively, report log_2_ fold-changes and -log_10_ of FDR-corrected *p*-values (-log (FDR)). Dotted line at -log_10_ 0.05. DESeq2 was utilized for DE analysis. Male saline *n* = 6, male morphine *n* = 7 rats, female saline *n* = 7, female morphine *n* = 3.

Analysis of samples of lumbar SCs stained against NeuN demonstrated a decrease in the area of staining in males on Day 6 (*p* < 0.05); no change was seen on Day 14 or on either day in females (*p* > 0.05) with morphine animals compared to same day saline controls (Figure 5). Representative images are found in Figure 5.

**Figure 6.**
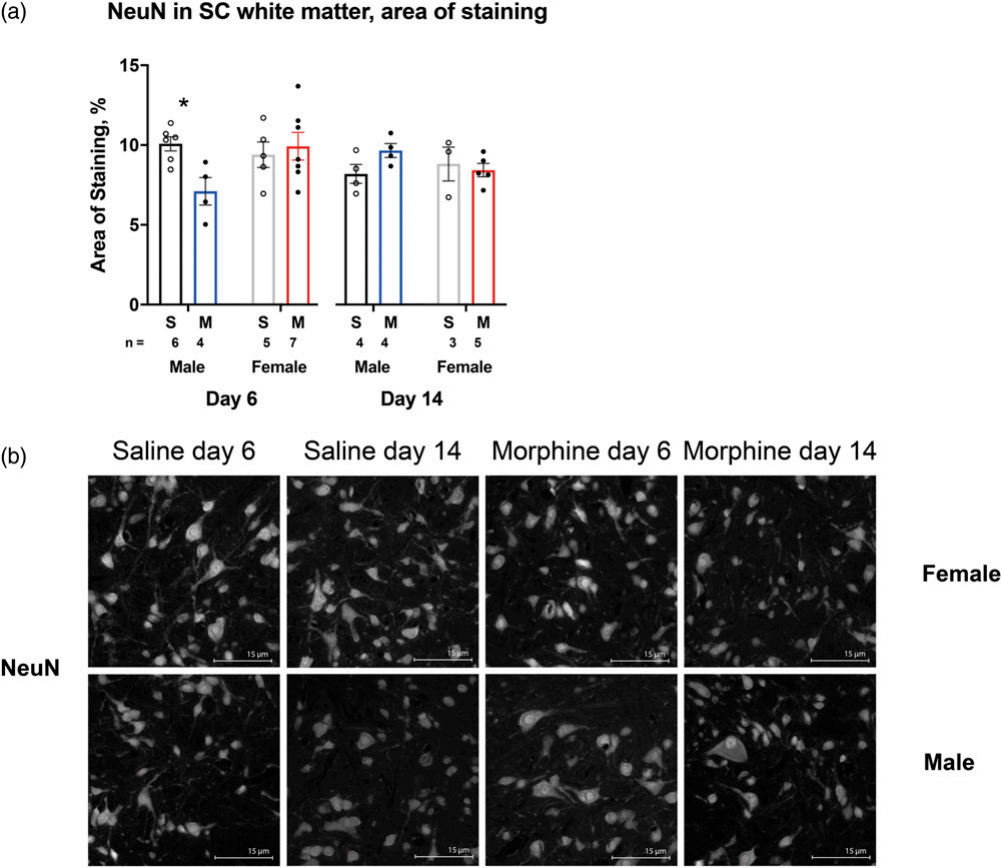
Analysis of NeuN-positive cells in the white matter of L4 spinal cord (SC) of male and female rats following chronic morphine administration on Days 6 (up to 30 mg/kg s.c.) and 14 (up to 50 mg/kg s.c.). The mean area of staining cells in % and SEM are reported in (a) and representative figures shown in (b). The entire area of the representative images was analysed. Same-day morphine (M) and saline (S) groups of the same sex were compared. *p < 0.05, Student’s *t* test; Male saline day 6 *n* = 6, Male morphine day 6 *n* = 4, Female saline day 6 *n* = 5, Female morphine day 6 *n* = 7, Male saline day 14 *n* = 4, Male morphine day 14 *n* = 4, Female saline day 14 *n* = 3, Female morphine day 14 *n* = 5.

### Microglial RNA expression

**Figure 7.**
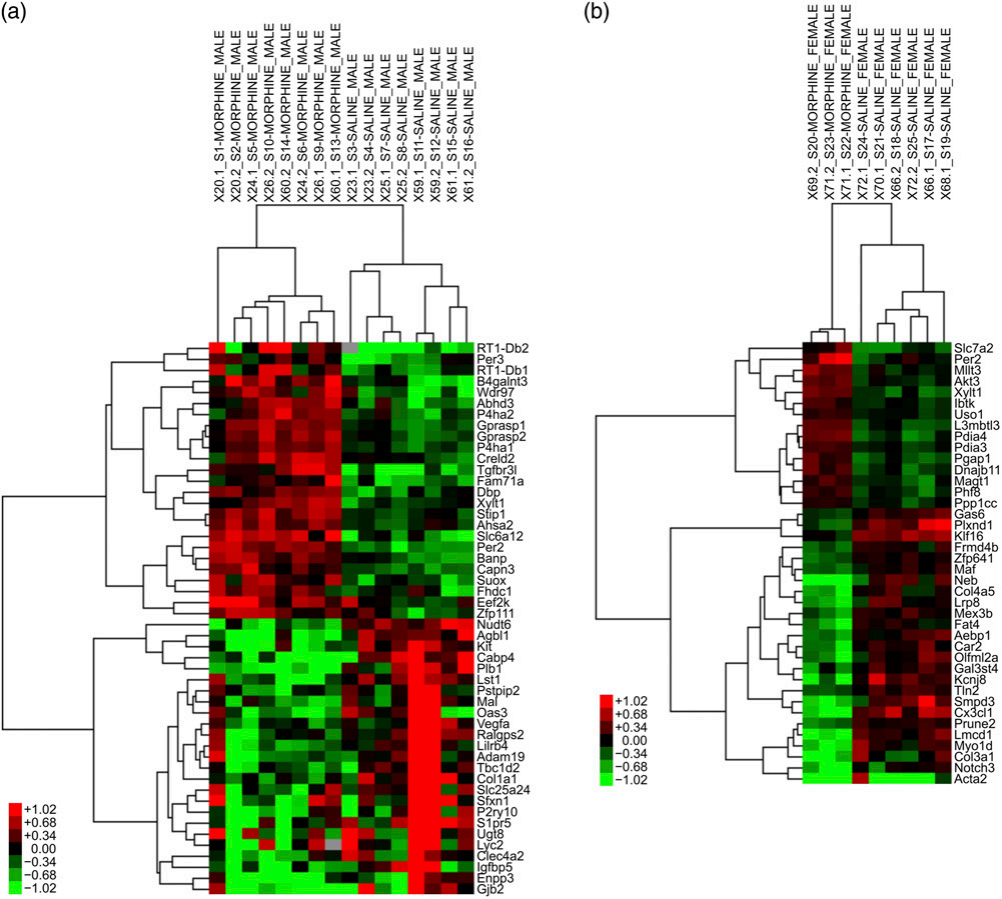
Heat maps of differentially expressed genes in rat spinal cord microglia following chronic high-dose morphine administration, male and female results are reported in ((a) and (b)), respectively.

In total, 509 and 52 transcripts in microglial cells showed altered expression in male and female rats, respectively, following chronic morphine treatment, compared with the respective saline groups (FDR-corrected *p*-value <0.05) (Supplementary Tables 1 and 2). This is likely due to the smaller group size (3 rats) in the female morphine group affecting the statistical analyses. Of these, 21 genes were differentially expressed in both sexes, while 488 and 30 were altered exclusively in males and females, respectively. Volcano plots of the transcriptomic data is visualized in Figure 6 and heat maps in Figure 7. The 25 genes demonstrating the largest up- and downregulation (FC) are listed in Tables 1 and 2, with only 15 genes being upregulated in females.

**Table 1.** Log_2_-fold-change (FC) and False Discovery rate (FDR) of 25 genes (15 in females) with the largest upregulation in gene expression per log_2_ FC in female and male microglia from rat L4-L5 spinal cord after 10 days of chronic morphine administration, compared with saline: Male saline *n* = 6, male morphine *n* = 7, female saline *n* = 7, female morphine *n* = 3. DESeq2 was utilized for the differential expression analysis.

Protein-coding genes with the largest upregulation in L4-L5 spinal cord microglia					
Male morphine versus male saline			Female morphine versus female saline		
Gene name	log_2_ FC	Adjusted *p*-value	Gene name	log_2_ FC	Adjusted *p*-value
*RT1-Db2*	2.74	1.96^E^-03	*Per2*	0.87	9.76E-04
*Tgfbr3l*	1.31	9.03^E^-03	*Pdia4*	0.69	7.59^E^-03
*Slc6a12*	1.23	2.08E-07	*Xylt1*	0.68	2.68E-02
*B4galnt3*	1.20	3.84E-02	*Slc7a2*	0.64	3.49E-02
*Per2*	1.15	1.18E-16	*L3mbtl3*	0.59	5.14^E^-03
*Wdr97*	0.91	2.84E-02	*Mllt3*	0.55	1.14E-02
*Banp*	0.86	8.92E-07	*Pgap1*	0.52	2.68E-02
*Abhd3*	0.85	1.08E-02	*Dnajb11*	0.51	1.04E-02
*Fam71a*	0.79	9.96E-04	*Akt3*	0.48	2.68E-02
*Gprasp1*	0.78	2.92E-07	*Magt1*	0.44	2.30E-02
*Dbp*	0.77	4.59E-05	*Phf8*	0.41	2.30E-02
*RT1-Db1*	0.77	2.37E-02	*Pdia3*	0.36	3.90E-02
*Suox*	0.77	5.50^E^-03	*Ibtk*	0.36	1.71E-02
*Capn3*	0.77	3.32^E^-03	*Uso1*	0.30	1.22E-02
*Stip1*	0.75	1.14E-06	*Ppp1cc*	0.27	4.44E-02
*P4ha2*	0.74	1.42^E^-03	—	—	—
*Gprasp2*	0.74	1.15E-05	—	—	—
*Creld2*	0.72	3.69E-04	—	—	—
*Xylt1*	0.70	1.06E-05	—	—	—
*Per3*	0.70	7.86^E^-03	—	—	—
*Eef2k*	0.69	2.82^E^-03	—	—	—
*Zfp111*	0.67	2.12^E^-03	—	—	—
*P4ha1*	0.66	2.80E-06	—	—	—
*Ahsa2*	0.66	1.15E-05	—	—	—
*Fhdc1*	0.65	2.71E-02	—	—	—

**Table 2.** Log_2_ fold-change (FC) and FDR of 25 genes with the largest downregulation in gene expression per log_2_ FC in female and male rat microglia from L4-L5 spinal cords after 10 days of chronic morphine administration, compared with saline: Male saline *n* = 6, male morphine *n* = 7 rats, female saline *n* = 7, female morphine *n* = 3. DESeq2 was utilized for the differential expression analysis.

Protein coding genes with the largest downregulation in L4-L5 spinal cord microglia					
Male morphine versus male saline			Female morphine versus female saline		
Gene name	log_2_ FC	Adjusted *p*-value	Gene name	log_2_ FC	Adjusted *p*-value
*Lyc2*	−2.40	4.16E-02	*Neb*	−2.14	1.47E-02
*Ugt8*	−2.18	2.68E-02	*Cx3cl1*	−1.96	5.14^E^-03
*Sfxn1*	−2.14	4.27E-02	*Acta2*	−1.96	1.09E-02
*Gjb2*	−2.07	2.45E-02	*Smpd3*	−1.55	6.39E-03
*Oas3*	−1.89	2.51E-02	*Lmcd1*	−1.36	3.99E-02
*Col1a1*	−1.86	6.78^E^-03	*Col3a1*	−1.34	5.84^E^-03
*Enpp3*	−1.85	2.04E-02	*Kcnj8*	−1.27	2.30E-02
*P2ry10*	−1.83	2.24E-02	*Fat4*	−1.14	2.68E-02
*S1pr5*	−1.83	1.13E-02	*Myo1d*	−1.06	1.04E-02
*Igfbp5*	−1.78	3.03E-02	*Notch3*	−1.03	6.04E-03
*Plb1*	−1.73	1.40^E^-03	*Plxnd1*	−1.00	4.78E-02
*Tbc1d2*	−1.56	1.55E-02	*Olfml2a*	−0.98	2.68E-02
*Clec4a2*	−1.53	2.93E-02	*Lrp8*	−0.91	2.00E-02
*Adam19*	−1.53	4.29E-02	*Col4a5*	−0.85	1.04E-02
*Lilrb4*	−1.51	2.44E-02	*Car2*	−0.75	3.26E-03
*Ralgps2*	−1.48	1.96E-02	*Aebp1*	−0.75	7.59^E^-03
*Mal*	−1.46	8.35^E^-03	*Mex3b*	−0.60	5.84^E^-03
*Lst1*	−1.37	7.84^E^-03	*Klf16*	−0.59	1.27E-03
*Slc25a24*	−1.34	2.91E-02	*Prune2*	−0.58	8.92^E^-03
*Vegfa*	−1.32	1.54E-02	*Gal3st4*	−0.58	4.51E-02
*Cabp4*	−1.31	3.93E-02	*Tln2*	−0.51	1.04E-02
*Kit*	−1.29	4.04E-02	*Maf*	−0.50	1.04E-02
*Agbl1*	−1.28	3.96E-04	*Frmd4b*	−0.50	2.30E-02
*Nudt6*	−1.27	3.67E-02	*Zfp641*	−0.48	7.59^E^-03
*Pstpip2*	−1.26	3.70E-02	*Gas6*	−0.48	1.04E-02

**Figure 8.**
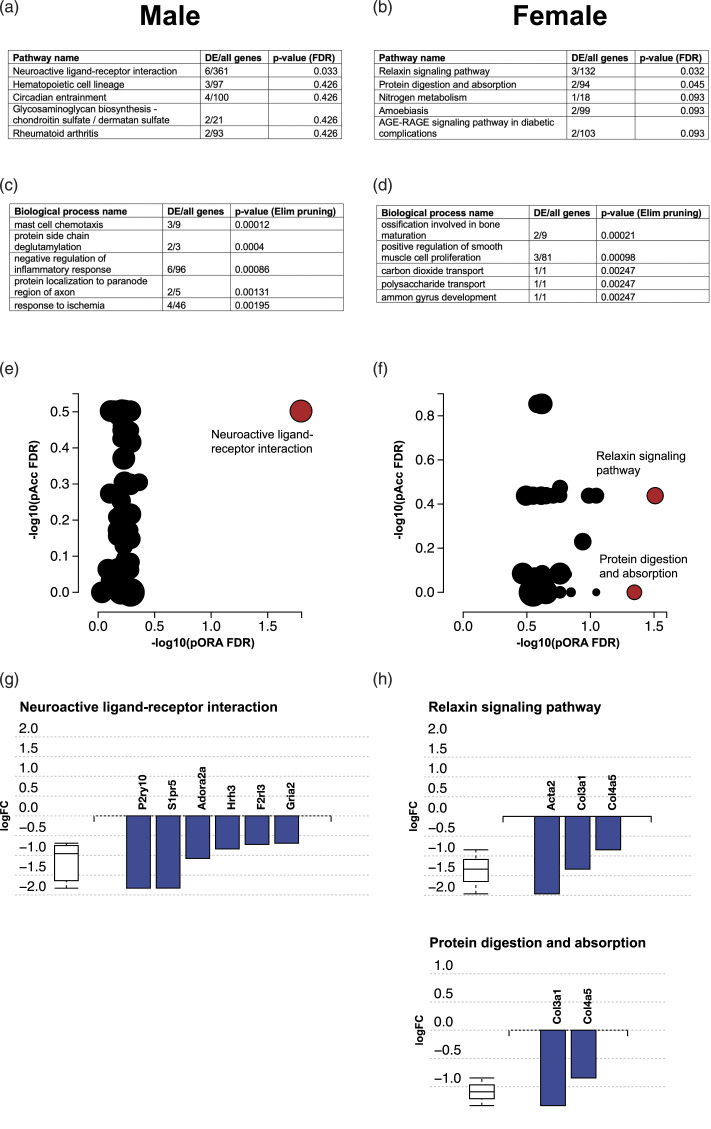
Pathways (KEGG)^49^ most affected in microglia of male rats from lumbar spinal cords following 10 days of chronic morphine administration. The five most significantly affected pathways in males and females are shown in ((a) and (b)), respectively, with the number of differentially expressed genes, total genes in pathway and FDR-corrected *p*-values reported. The five most significantly affected biological processes in males and females are reported in ((c) and (d)), respectively. Overrepresentation and accumulation of genes in the pathways are reported in ((e) and (f)). All genes with differential expression in the significantly affected pathways (FDR corrected *p*-value <0.05) are reported in ((g) and (h)). pORA = overrepresentation, pAcc = total pathway accumulation, DE genes = differentially expressed genes. Dots represent pathways, significantly affected pathways are red and non-significant are black. The size of each dot is proportionate to the pathway size. Male saline *n* = 6, male morphine *n* = 7, female saline *n* = 7, female morphine *n* = 3. Analyses and graphs were created with iPathwayGuide from AdvaitaBio.

iPathwayGuide analyses of the microglial RNA-Seq data after morphine revealed few affected pathways. In males, only the “Neuroactive ligand-receptor interaction” pathway was affected (FDR = 0.033). In females, “Relaxin signalling pathway” (FDR = 0.032) and “Protein digestion and absorption” (FDR = 0.045) were affected by the morphine treatment (Figure 8). Several biological processes were affected by the treatment, of which the most significant are reported in Figure 8.

In addition, we compared differences in gene expression of microglia in males and females in the saline groups. In saline-treated animals, 1136 genes showed sexually dimorphic expression (adjusted *p*-value <0.05). The 25 most differentially up- and downregulated genes per log_2_ FC when comparing male and female saline animals are shown in Table 3.

**Table 3.** Log_2_ fold-change (FC) and FDR of 25 genes with the largest up- and downregulation in gene expression per log_2_ FC in male microglia from rat L4-L5 spinal cords (SC) treated with saline, compared with female saline group microglia: Male saline *n* = 6, female saline *n* = 7. DESeq2 was utilized for the DE (differential expression) analysis.

Male saline versus female saline L4-L5 spinal cord microglial gene expression		
Gene name	log_2_ FC	Adjusted *p*-value
*Ddx3*	13.62	5.71E-56
*Kdm5d*	11.32	2.87E-38
*Eif2s3y*	10.30	2.51E-31
*Lin28a*	3.53	3.40E-02
*AABR07015078.1*	2.57	2.08E-02
*Slc22a15*	2.52	2.94E-02
*Cenpu*	2.34	2.87E-02
*AABR07063424.1*	2.18	3.10E-04
*Rpl30*	2.06	2.25E-02
*Ugt8*	2.06	4.36E-02
*Pln*	1.92	1.15E-02
*Pcp4*	1.90	2.27E-02
*Lmod1*	1.37	3.57E-02
*Sema6b*	1.37	4.78E-02
*LOC500035*	1.33	1.18E-02
*Rab26*	1.33	1.89E-02
*Tmem126b*	1.29	3.19E-03
*LOC103692716*	1.26	3.43E-02
*RGD1560821*	1.26	4.06E-02
*Dach1*	1.24	1.71E-02
*Zbtb16*	1.24	2.54E-02
*Hsp90aa1*	1.19	4.01E-05
*LOC362863*	1.14	3.38E-02
*Nefm*	1.13	1.01E-03
*Immp1l*	1.09	3.35E-02
*Ednrb*	−0.92	1.43E-02
*Itga5*	−0.94	6.92E-13
*Map3k6*	−0.96	5.31E-03
*Msantd1*	−0.98	4.50E-03
*LOC100359777*	−0.99	2.86E-02
*Mfsd6l*	−1.00	3.39E-02
*Lmna*	−1.01	2.86E-07
*Sdc4*	−1.04	1.66E-02
*Ckap2l*	−1.06	3.56E-04
*Il22ra2*	−1.07	2.55E-03
*Pnma1*	−1.14	9.52E-03
*Tm4sf19*	−1.14	9.13E-03
*Tgfbr3l*	−1.14	2.86E-02
*Ccdc40*	−1.15	5.21E-04
*Gpr3*	−1.18	2.24E-03
*Folr2*	−1.24	4.32E-03
*Grhl1*	−1.30	2.57E-03
*Kcnk7*	−1.40	7.46E-03
*Mybpc3*	−1.43	1.74E-13
*Mlf1*	−1.58	6.81E-05
*AC120291.4*	−1.81	2.89E-04
*LOC680227*	−1.86	4.21E-12
*LOC290876*	−2.08	3.47E-03
*RT1-Db2*	−2.44	9.32E-03
*Fam186b*	−2.82	1.29E-02

## Discussion

### Main findings

We analysed antinociception and tolerance in male and female rats during chronic high-dose morphine administration using a thermal assay, IHC/IF markers of microglia and neurons, and the transcriptome of microglia in the spinal cord. Similar antinociceptive tolerance developed to morphine in both sexes. We saw a slight decrease in males and females in the area of IBA1-positive staining cells following morphine treatment. The microglial transcriptome revealed several changes related to circadian rhythms, immunology, and apoptosis, among others.

### Antinociception and tolerance

Chronic morphine administration caused similar thermal antinociceptive tolerance to acute morphine in both sexes. Nor did we see any sex differences in the development of tolerance, or in the effect of morphine on thermal nociception in tolerant rats. Many studies point to a larger antinociceptive effect of acute morphine in male rats,^50–52^ and this difference has been attributed, among other factors, to differences in morphine metabolism in the CNS.^53^ However, while Holtman et al. demonstrated a larger antinociceptive effect of morphine in opioid-naïve males, they found no differences in antinociceptive effects of acute administration of morphine in tolerant male and female rats, (10–25 mg/kg), with similar tolerance in both sexes.^54^

### Effects on microglial and neuronal staining

We demonstrated a decrease in IBA1-positive staining cells following high-dose opioid administration in both sexes, when compared with saline control groups. In the CNS, IBA1 is a quite specific marker for microglia.^55^ Interestingly, prior studies have consistently shown an increase in IBA1-positive cell staining. The observed decrease in our study is thus novel finding. The prior studies used smaller doses, which might explain the difference.^19,39,56^ For example, our group previously used morphine 10 mg/kg s.c. twice daily and saw a 33% increase in spinal dorsal horn IBA1-positive cell staining.^39^ In this study, we used larger doses of morphine, rising to 50 and 40 mg/kg s.c. twice daily. Interestingly, Jokinen et al. did observe a decrease in the IBA1 positive staining cells in the microglia-rich substantia nigra. Morphine administration is known to cause oxidative stress,^25,28^ and in vitro, morphine has been shown to cause apoptosis in microglia and neurons.^31^ In addition, fractalkine (CX3CL1) inhibits Fas-mediated apoptosis of microglia,^57^ and was decreased in our RNA-Seq data. We hypothesize that chronic large doses of morphine have an activation-limiting, or toxic effect in microglia, but the mechanisms and critical dosing need to be studied further.

We also demonstrated a slight decrease in NeuN positive cell -staining, a neuronal marker, in the white matter of the SC, but only in males, and only on day 6. Chronic high-dose oxycodone has been shown to activate pro-apoptotic pathways in the white matter of rat brains.^29^ The shorter treatment in our study, compared with 30 days in the study of Fan and co-authors, and the fact that we analysed samples from the spinal cord may explain why no change was seen on day 14. To conclude, the effects of long-term high-dose opioids on neurons in the SC is unclear based on these results.

### Effects on the transcriptome

In our RNA-Seq data of male and female microglia, we discovered several sex differences following treatment with high doses of morphine and in the saline groups. Our most important findings regard genes related to circadian rhythms, inflammation, and apoptosis. The much smaller group size in the female morphine group greatly impair our ability to compare results for the two sexes. Previous analyses of the microglial transcriptome in mice, however, also display stronger acute inflammatory responses in males after peripheral nerve injury, and several sexually dimorphically expressed genes.^58,59^

#### Circadian rhythms

Circadian rhythms and sleep are of paramount importance for health in general and in chronic pain,^60^ and as detailed in the introduction, microglia may link these phenomena. Several genes regulating circadian rhythms have important roles in neuropathic and inflammatory pain^61,62^ and in OIH.^17^ Among others, the product of the *Clock* gene drives *Tac1* expression, which in turn directly upregulates Substance P, an excitatory neuropeptide, in dorsal root ganglia.^63^ Importantly, fentanyl has been shown to decrease *Per1* gene expression during night, and inconclusively affect *Per2*.^64^ Both *Clock* and *Per1 are k*ey genes in the regulation of the circadian rhythm.^65^

As such, it is interesting, that *Per2* was the most significantly upregulated gene in females and among the most upregulated genes in males in our study. Furthermore, *Per3,* another regulator of the sleep-wake cycle,^66^ was upregulated in males. *Clock* expression, however, was not affected. In addition, *Dbp* (D site of albumin promoter (albumin D-box) binding protein), a gene oscillating with a circadian rhythm,^67^ was upregulated in males. Previous studies have demonstrated circadian rhythmicity in microglia, for example, in microglial levels of interleukins (IL) 1β and 6, and TNFα. Intriguingly, glucocorticoids induce *Per1* expression, while acute morphine also affects *Per1* expression.^15^ Furthermore, acute morphine has been shown to shift the time phase in the suprachiasmatic nucleus, and to affect *Per1* but not *Per2*.^15^

This suggests a link between circadian and inflammatory functions.^20^ Disruptions in the microglial circadian clock have also been linked to neurodegenerative diseases such as Alzheimer’s disease.^68^

#### Inflammation

*Ccr3*, the gene encoding for a chemokine receptor which binds many different chemokines, was downregulated in both sexes (log_2_ FC in males: −1.18; and in females: −0.833). Interestingly, a previous study of an Alzheimer’s disease model has shown a link between reduced cell surface CCR3 and reduced microgliosis, in line with our results.^69^

We also identified, in males only, an upregulation of *RT1-Db2*, a component of MHC class II, which is important for antigen presentation.^70^ Additionally, we analysed saline group differences between the sexes. Interestingly, males had lower interleukin one beta expression (log_2_ FC –0.65).

Classically, microglial cells are categorised as M1 or M2. The M1 phenotype produces high levels of pro-inflammatory cytokines and reactive oxygen species and is paramount in antimicrobial defence; the M2 phenotype is central for the regulation of immune responses, tissue remodelling, and antiparasitic activities.^71^ To investigate microglial polarisation following treatment with morphine, we analysed possible markers of polarisation to the M2 phenotype.^72,73^ The slight upregulation of interleukin one beta in males (log_2_ FC 0.29) might imply M1 polarisation, as would downregulation of vascular endothelial growth factor A (*Vegfa*) in males (log_2_ FC –1.32), a marker linked to M2 cells. Further, *Tnfsf10,* a member of the tumour necrosis factor alpha (TNFα) family*,* associated with M2 cells, was also downregulated in males (log_2_ FC –0.85). However, *Tgfbr3l*, a type of transforming growth factor linked to M2 cells, was upregulated (log_2_ FC 1.32). We also analysed C-C motif chemokines, genes of great importance in microglial polarisation. However, none showed changes following treatment, as did none of the differentiation genes relevant for polarisation. We conclude that no major microglial polarisation occurred following chronic high-dose morphine treatment. Jokinen et al. have previously analysed the transcriptome of male spinal microglia and described M2-polarisation of microglia.^39^ The differences in dosing may explain this different result.

#### Apoptosis

*Cx3cl1,* which encodes fractalkine (CX3CL1), was downregulated in both sexes (log_2_ FC: M −0.98 and F −1.96) and has several roles in microglial function. CX3CL1 binds to CX3CR1 receptors, and acts both as a chemoattractant,^74,75^ and an enhancer of microglial effector functions, including phagocytosis,^76,77^ also inhibiting apoptosis of microglia.^57^ As such, this downregulation, together with observed decreased IBA1-staining, is intriguing. However, as CXC3L1 is largely released by neurons and bound by microglial CXC3R1, the implications of its downregulation in microglial expression are unclear.^78^

*Capn3*, which encodes calpain3, important in astrocyte plasticity and muscle diseases, was upregulated in males. It has also been identified as an important protease in apoptosis, leading to membrane degradation and ultimately to cell death.^79,80^

*Lyc2*, encoding LYC2, a lycopene, showed one of the largest downregulations in males. It is considered a potent anti-oxidant, anti-apoptotic protein. It alleviates H_2_O_2_-induced oxidative stress, and downregulates the caspase pathway.^81,82^

Moreover, *Stip1*, another gene that was upregulated solely in males, has also been shown to induce apoptosis.^83^ Together, these changes in microglial gene expression demonstrate possible links between the decreased IBA1-staining and apoptosis in microglia.

#### Other

Chronic opioid treatment upregulated *Slc6a12*, which encodes a GABA transporter, in male rats. An inhibition of the release of GABA through presynaptic MORs reduces GABA-mediated disinhibition of the descending inhibitory pain circuits.^1^ It remains to be shown if long-term opioid treatment can modulate GABAergic neurotransmission through these novel mechanisms.

Of interest is also *ApoE*, which was downregulated in males (log_2_ FC = −0.34). *ApoE* is of importance in lipid metabolism, but also, importantly, has been proposed as a key mediator in chronic pain in microglia following a traumatic peripheral nerve injury.^58^ Tansley et al. demonstrated upregulation of the gene in microglia, while we showed decreased expression after morphine; however, whereas they reported increased microgliosis, we demonstrated a decrease. This additionally underlines, that chronic high-dose morphine, causes distinct changes in microglia, compared to several other treatment models.

## Conclusions

We demonstrated a similar development of opioid antinociception and tolerance with high doses of morphine in male and female rats. Immunohistochemistry revealed a decrease in IBA1-positive cell staining in spinal cords following treatment in both sexes. The transcriptomic data from SC microglia revealed several changes in circadian and inflammatory genes. These results provide further support for the convergence in the functions of microglia, the sleep-wake cycle and apoptosis. Our results suggest that there may be under-recognised long-term effects of high doses of opioids which may have important clinical implications in patients receiving chronic high-dose opioid treatment.

## Supplemental Material

Supplemental Material - The effects of chronic high-dose morphine on microgliosis and the microglial transcriptome in rat spinal cord Click here for additional data file. Supplemental Material for The effects of chronic high-dose morphine on microgliosis and the microglial transcriptome in rat spinal cord by Fredrik HG Ahlström, Hanna Viisanen, Leena Karhinen, Kert Mätlik, Kim J Blomqvist, Tuomas O Lilius, Yulia A Sidorova, Vinko Palada, Pekka V Rauhala and Eija A Kalso in Molecular Pain

Supplemental Material - The effects of chronic high-dose morphine on microgliosis and the microglial transcriptome in rat spinal cord Click here for additional data file. Supplemental Material for The effects of chronic high-dose morphine on microgliosis and the microglial transcriptome in rat spinal cord by Fredrik HG Ahlström, Hanna Viisanen, Leena Karhinen, Kert Mätlik, Kim J Blomqvist, Tuomas O Lilius, Yulia A Sidorova, Vinko Palada, Pekka V Rauhala and Eija A Kalso in Molecular Pain
